# Case Report: Exome Sequencing Identified a Novel Compound Heterozygous Variation in *PLOD2* Causing Bruck Syndrome Type 2

**DOI:** 10.3389/fgene.2021.619948

**Published:** 2021-02-16

**Authors:** Jing Zhang, Huaying Hu, Weihong Mu, Mei Yu, Wenqi Chen, Dongqing Mi, Kai Yang, Qing Guo

**Affiliations:** ^1^Prenatal Diagnosis Center, Shijiazhuang Obstetrics and Gynecology Hospital, Shijiazhuang, China; ^2^School of Medicine, Xiamen University, Xiamen, China; ^3^Jiaen Genetics Laboratory, Beijing Jiaen Hospital, Beijing, China; ^4^Prenatal Diagnosis Center, Beijing Obstetrics and Gynecology Hospital, Capital Medical University, Beijing, China

**Keywords:** *PLOD2* gene, Bruck syndrome type 2, osteogenesis imperfecta, whole-exome sequencing, immunohistochemistry detection

## Abstract

Bruck Syndrome (BRKS) is a rare type of recessive osteogenesis imperfecta (OI) and consists of two subtypes, BRKS1 and BRKS2, which are caused by variations in *FKBP10* and *PLOD2* genes, respectively. In this study, a family that had experienced multiple miscarriages and recurrent fetal skeletal dysplasia was recruited for the purpose of a multiplatform laboratory investigation. Prenatal genetic testing with whole-exome sequencing (WES) identified a compound heterozygous variation in the *PLOD2* gene with two variants, namely c.2038C>T (p.R680^*^) and c.191_201+3 delATACTGTGAAGGTA (p.Y64Cfs^*^12). The amino acids affected by the two variants maintained conserved across species. And the result of immunohistochemistry (IHC) indicated that the expression of PLOD2 protein in the proband's osteochondral tissue was significantly decreased. These findings in our study expanded the variation spectrum of *PLOD2* gene, provided solid evidence for the family's counseling in regard to future pregnancies, strongly supported the application of WES in prenatal diagnosis, and might give insight into the understanding of PLOD2 function.

## Introduction

Congenital skeletal dysplasia (SD) often displays severe *in utero* manifestations, which provides clues and challenges for prenatal diagnosis (Offiah, 2015; Liu et al., 2019). First, they can provide evidence for the timely formation of management plans; on the other hand, due to the strong clinical heterogeneity of SDs, it is difficult to accurately judge the prognosis by these indeterminate indications alone (Konstantinidou et al., 2009; Offiah, 2015). Under such circumstances, a meticulously designed strategy of genetic detection would help overcome the setbacks in clinical differential diagnosis (Zhou et al., 2018; Yang et al., 2019).

Osteogenesis imperfecta (OI) is a series of congenital metabolic bone disorders and comprises dozens of conditions ranging from early lethality to mild manifestations (Marini, 2013; Liu et al., 2017). OI is mainly characterized by increasing bone fragility, recurrent fracture, and subsequent growth retardation (Forlino and Marini, 2016; Liu et al., 2017). OI patients also share some extraskeletal symptoms, such as blue sclera, hearing deficits, dentinogenesis imperfecta (DI), and valvular heart disease (Forlino and Marini, 2016). Bruck syndrome types 1 and 2 (BRKS1, MIM #259450; BRKS2, MIM #609220), caused by biallelic pathogenic variants in the*FKBP10* (MIM ^*^607063) and *PLOD2* (MIM ^*^601865) genes, respectively, are two rare subtypes of OI, mainly characterized by congenital joint contractures and pterygia other than the common OI manifestations (Viuoen et al., 1989). *PLOD2* encodes lysyl hydroxylase 2 (LH2, EC 1.14.11.4), while *FKBP10* encodes the prolyl cis-trans isomerase FKBP65, the activities of which are required in forming mature crosslinks in bone collagen (Bank et al., 1999; Alanay et al., 2010).

To our knowledge, fewer than 20 studies have reported the BRKS2 cases with respect to defects in *PLOD2* (Mumm et al., 2020). Moreover, the genotype-phenotype correlation of BRKS2 is not fully established, owing to the low amount and relatively clustered distribution of constitutional variations in *PLOD2*, the vague structural and functional delineation of the N-terminal of LH2 protein as well as the strong phenotypic variability of BRKS2 (Tham et al., 2018; Mumm et al., 2020). Furthermore, given the commonality of cell signaling pathways between BRKS2 and multiple types of cancers, the in-depth study of corresponding molecules would benefit scientific and clinical understanding in both fields (Guo et al., 2018; Wang et al., 2020).

Thus, we studied a family having experienced multiple abnormal gestations. Prenatal ultrasonography examination and genetic detections were conducted to identify the cause of fetal skeletal dysplasia in the subject family.

## Case Presentation

A 34-year-old woman was first referred to our Center in February 2015 for having experienced multiple abnormal gestations. At that point she was 8 weeks pregnant.

**Figure 1:**
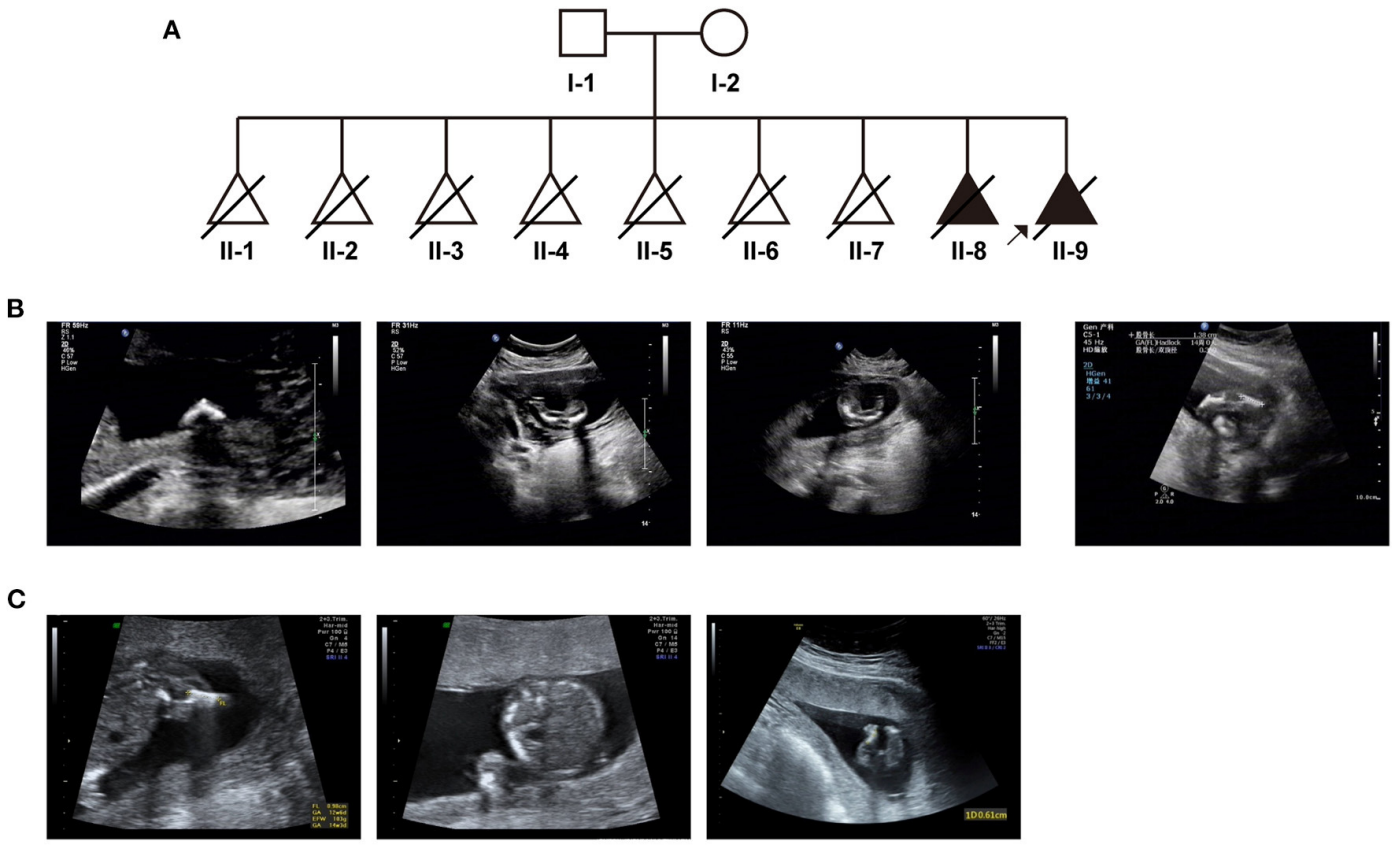
The clinical findings in this case: **(A)** Pedigree diagram of the family with fetal skeletal dysplasia and multiple miscarriages. **(B)** Ultrasonographic indications of the couple's eighth pregnancy. **(C)** Ultrasonographic indications of the couple's most recent (ninth) pregnancy.

Based on medical records and her personal dictation, we combed through the couple's complete medical history and illustrated in a pedigree diagram (Figure 1A). They had eight previous pregnancies in total: In August 2005, missed abortion occurred at the eighth gestational week of their first pregnancy. In October 2006, it was diagnosed that the second pregnancy was an ectopic pregnancy on the left fallopian tube, whereupon transabdominal resection of the left fallopian tube was performed. In October 2007, July 2008, August 2010, and December 2012, the couple went through four missed abortions or miscarriages, all of which occurred between 8 and 16 gestational weeks. In November 2013, the fetus of their seventh pregnancy was diagnosed with thickened nuchal translucency (NT), right abdominal fissure and visceral valgus. Induced labor was performed at 15 weeks of gestation.

In 2015, all indicators of their 8th pregnancy during early pregnancy were normal, including serological screening, NIPT (non-invasive prenatal testing) results. However, at 17th week of gestation, ultrasonic examination revealed that the fetus had extremely short limbs and bowing of long bones (Figure 1B). Amniocentesis and subsequent prenatal diagnosis with chromosomal karyotyping and micro array (CMA) were conducted. However, no variation with clinical significance was identified. The fetus was aborted at 20 gestational weeks.

In April 2018, we performed prenatal diagnosis for their most recent pregnancy at the 16th week of gestation. Ultrasonography indicated that bilateral lower limbs of the fetus were shortened, not excluding the presence of bending (Figure 1C). Besides regular prenatal genetic testing as described above, whole-exome sequencing was also introduced after induction.

## Laboratory Investigations

Prenatal genetic testing including karyotyping and CMA (with Cytoscan 750k, Thermo Fisher platform) were performed on the last two adverse pregnancies in 2015 and 2018. Both results of those were normal.

**Figure 2:**
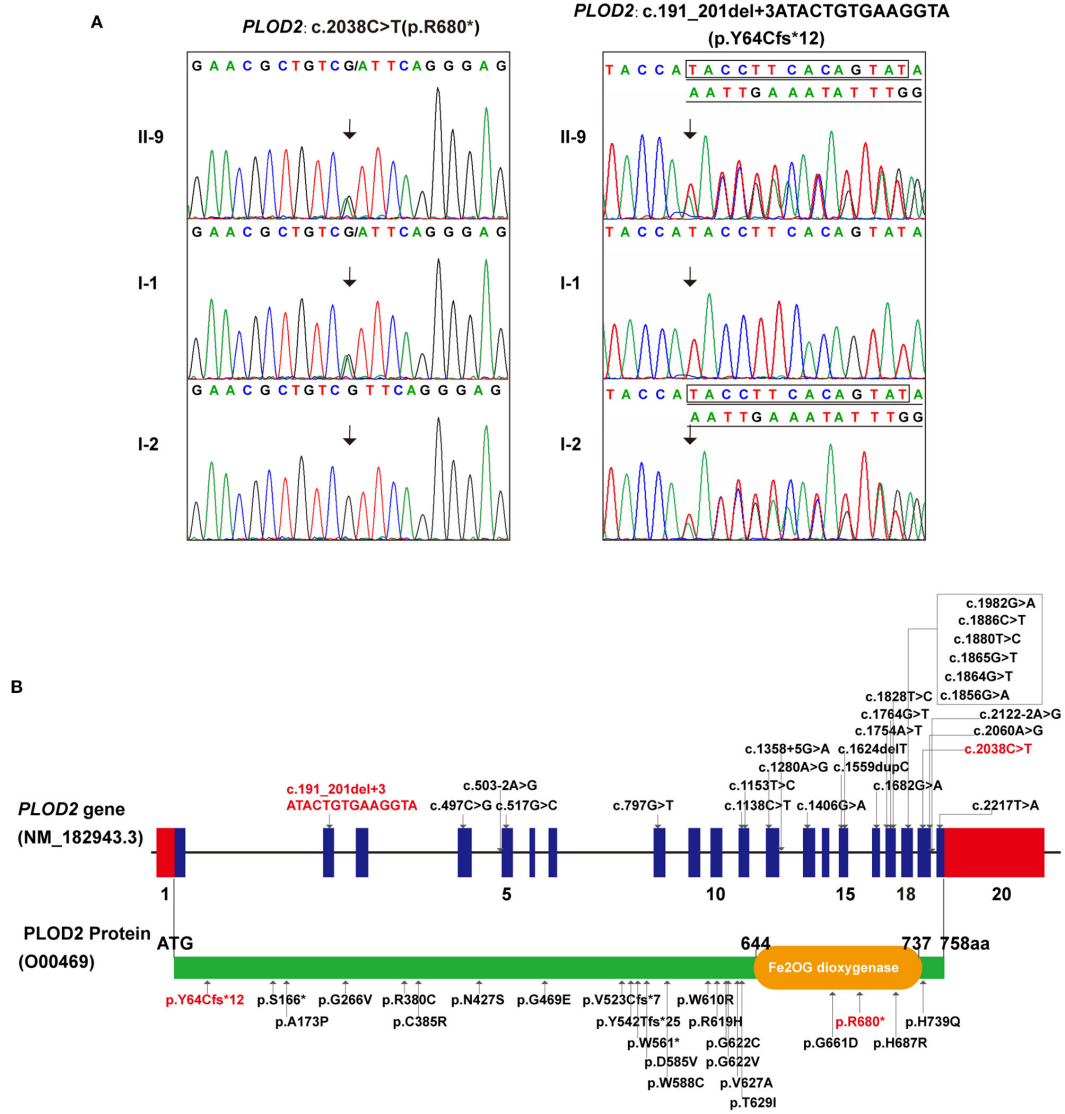
The genetic variations identified in this case: **(A)** The two variants detected in this fetus and his parents (individual no. corresponding to Figure 1A). **(B)** All *PLOD2* variants associating with BRKS2-like phenotype in the literature and in this study, illustrated in diagrams of gene and protein. (In this study, red font represents variants).

Accordingly, whole-exome sequencing was performed on the skin tissue obtained from the aborted fetus of the last pregnancy. In brief, the enrichment of target-region sequences was performed by means of the Sure Select Human Exon Sequence Capture Kit (Agilent, USA). The sequencing libraries were quantified using the DNA Standards and Primer Premix Kit (Kapa Biosystems, USA), massively parallel-sequenced using the XTEN platform and then massively parallel-sequenced again (Illumina, USA). After sequencing and filtering out the low-quality readings, the high-quality readings were compared to the human genome reference sequence [(hg19/GRC h37)]. The GATK software (Genome Analysis TK3.3.0, https://software.broadinstitute.org/gatk/) and the Verita Trekker® Variants Detection system (Berry Genomics, China) were used to identify single-nucleotide polymorphisms, insertion and deletions, copy-number variants (CNVs), mitochondrial gene variants, and runs of homozygosity. Subsequently, the Enliven® Variants Annotation Interpretation (Berry Genomics, China) system was used to fulfill the annotation and interpretation progress referring to multiple databases [1000g2015aug_eas (https://www.internationalgenome.org/); ExAC_EAS (http://exac.broadinstitute.org); gnomAD_exome_EAS (http://gnomad.broadinstitute.org/); HGMD®: Human Gene Mutation Database (Professional Version 2019.4)]. The suspected pathogenic variant was validated by Sanger sequencing through use of ABI 3730 Automated Sequencer (Applied Biosystems). The mutations were identified by sequence alignment with the NCBI Reference Sequence (NG 011537.1) using Chromas 2.33. WES identified a compound heterozygous variation in the *PLOD2* gene consisting of two variants, namely c.2038C>T (p.R680^*^) and c.191_201+3delATACTGTGAAGGTA (p.Y64Cfs^*^12) (NM_000935) (Figure 2A). Sanger sequencing revealed that the father carried the heterozygous c.2038C>T variant, while the mother carried c.191_201+3del (Figure 2A). The former one was reported by Lyu et al. as a pathogenic variant (Lv et al., 2017), while the latter, which would result in a premature truncated protein, was first reported in this study. According to the variant interpretation criteria by ACMG (Richards et al., 2015), it was classified as pathogenic, with the evidence of PVS1, PM2, PM3, and PP4.

**Figure 3:**
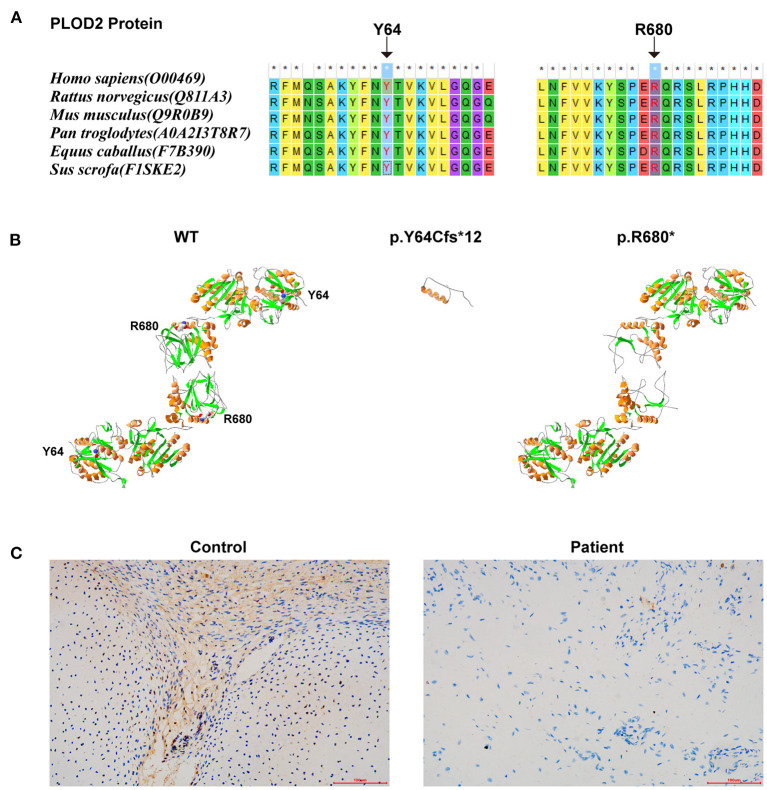
The results of *in silico* analysis and IHC experimentation: **(A)** The conservatism of the two amino acids effected by the two variants. **(B)** The protein models of WT, p.Y64Cfs*12, and p.R680* of *PLOD2* protein model 6fxm.1.A. **(C)** *PLOD2* protein expression in the osteochondrocytes of the proband and control samples, by IHC.

The conservatism of all amino acids effected by detected variants was analyzed using MEGA7 (http://www.megasoftware.net/previousVersions.php) with default parameters. The results showed that the amino acids Y64 and R680 (i.e., the two variants) maintained evolutionary conservatism among species (Figure 3A).

Protein modeling was conducted using SWISS-MODEL (https://swissmodel.expasy.org/) and Swiss-Pdb Viewer software (https://spdbv.vital-it.ch/disclaim.html#). PLOD2 protein model of wild type (WT)-6fxm.1.A (Seq Identity:59.26%/GMQE: 0.82/QMEAN: −0.67), p.Y64Cfs^*^12-6fxm.1.A (Seq Identity:57.80%/GMQE: 0.80/QMEAN: −0.78), and p.R680^*^-6fxk.1.A (Seq Identity:43.59%/GMQE:0.18/QMEAN: −0.87) were shown (Figure 3B). According to the results, these two variants would result in premature truncated peptides, which could cause severe damage to protein function. Immunohistochemistry (IHC) (with Anti-PLOD2/LH2 antibody, ab90088, Abcam, USA) was conducted on the paraffin sections of fetal auricular finger osteochondrocytes of the proband (along with a cartilage tissue sample from an aborted fetus (*PLOD2* wild-type) at similar gestational age as normal control). The results indicated that the expression level of *PLOD2* protein was significantly decreased in the osteochondral tissues of the proband (Figure 3C). The low protein expression of *PLOD2* was possibly because c.2038C>T (p.R680^*^) and c.191_201+3delATACTGTGAAGGTA would be expected to induce non-sensical mediated decay.

## Discussion

Recessive forms only account for <10% in OI disorders, yet the list of causative genes has been growing through recent years (Eyre and Weis, 2013). Additionally, with the defects in various proteins involved in bone formation, OIs display a broad range of phenotypic variability (Forlino and Marini, 2016). This promotes the challenge in prenatal diagnosis and management on fetal SDs with similar early manifestations. Therefore, it would appear to be more and more arbitrary to consider the *in utero* limb shortening and bowing of long bones as a collagen-related autosomal dominant condition.

As a rare but typical example of recessive OI, most cases of BRKS are distinguished by congenital joint contractures and pterygia, albeit with several exceptions (Tham et al., 2018; Mumm et al., 2020). Currently, two BRKS subtypes, BRKS1 and BRKS2, with almost identical phenotypes but different pathogenesis are recognized (van der Slot et al., 2003; Alanay et al., 2010). To our knowledge, 25 constitutional mutations in *PLOD2* associating with BRKS2-like phenotypes have been detected in fewer than 20 studies, showing significant C-terminal aggregation (Figure 2B; Supplementary Table 1) (Mumm et al., 2020). In this study, a compound heterozygous variation in the*PLOD2* gene with two variants, c.2038C>T and c.191_201+3del, was identified and confirmed. The former one, c.2038C>T, was once reported in BRKS2 patient (Lv et al., 2017); while the latter one, c.191_201+3del, was a novelly detected frame-shift variant causing early termination of protein translation. This finding expanded the variation spectrum of *PLOD2* gene. Additionally, Guo et al. have determined a 2Å crystal structure of the lysyl hydroxylase (LH) domain corresponding to human *PLOD2* amino acids 548–758, which provided sub-molecular insight into how specific variants affect the structure of LH2 (Guo et al., 2018). The genotype-phenotype correlation of *PLOD2* skeletal dysplasia is pending further elucidation along with the identification of more variations, particularly at the N-terminal (Leal et al., 2018).

LH2, cloned by Valtavaara et al. (1997), is encoded by PLOD2 gene, the defect of which is responsible for the onset of BRKS2. LH2 specifically catalyzes the hydroxylation of telopeptide lysyl residues in collagen, which is essential for the normal crosslinking of bone collagen (Bank et al., 1999; van der Slot et al., 2003). FKBP10, whose deficiency is causative for BRKS1, belongs to a group of proteins termed immunophilins and displays high binding affinity for the immunosuppressant drugs FK506 (Patterson et al., 2000). FKBP10 is thought to function as a collagen chaperone and to assist in collagen folding (Lietman et al., 2014). Subsequently, Chen et al. found that the peptidyl prolyl isomerase activity of FKBP10 positively modulated LH2 enzymatic activity and was critical for the formation of hydroxylysine-aldehyde derived intermolecular collagen crosslinks, which indirectly explained why phenotypes of BRKS1 and BRKS2 were so similar (Chen et al., 2017). LH2 has two splice forms–LH2a (short) and LH2b (long)–resulting from alternative splicing of *PLOD*2, with LH2b containing an additional exon 13 A encoding 21 amino acids (Gjaltema and Bank, 2017). The absence of LH2b has great impact on musculoskeletal tissues, particularly bone, as illustrated by mutations in PLOD2 (Gjaltema and Bank, 2017). LH2 activity is positively correlated with pyridinoline levels, which indicates its important role in the formation of collagen telopeptide-derived pyridinoline crosslinks (van der Slot et al., 2005; Gjaltema and Bank, 2017). In this study, the result of IHC indicated the extremely low expression of *PLOD2* in cartilage tissue of the proband, which should be the result of the variation he carried. Both mutations c.2038C>T (p.R680^*^) and c.191_201+3delATACTGTGAAGGTA would be possibly expected to induce nonsense mediated mRNA decay (NMD), whereby the low expression level of *PLOD2* could be responsible for the NMD. Further functional experiments are needed to explore the possible mechanism. However, we failed to detect the mRNA-level expression of *PLOD2* due to the degradation of RNA in the fibroblast sample, which is a flaw in our study and needs to be remedied by subsequent functional studies. From another angle, the protein modeling result revealed that both mutations possibly caused the peptide chain truncation, which clearly affected the dimerization and maturation process and even the presence of the protein. Besides, the evolutionary conservatism of the amino acids affected by the two variants further supported their pathogenicity. However, further functional experiments are necessary, not only to clarify the effect of each variant on the protein itself but also to understand the role of this effect in the overall process of osteogenic differentiation.

In practical terms, diagnostic report in this study was obtained before the fetal pregnancy entered the perinatal period (after 27^+6^ gestational weeks), which was supportive for the application of WES in the field of prenatal diagnosis.

For any future pregnancy of the proband in this study, the recurrent risk of Bruck syndrome 2 condition would be 25%. Given such circumstances, the couple were informed of reproductive options such as prenatal testing and preimplantation genetic diagnosis (PGD). We need to follow up on the outcome of the couple's pregnancy.

## Conclusion

In summary, this study detected a novel *PLOD2* compound heterozygous variation in a 17-gestation-week fetus with BRKS2 by a multi-platform genetic approach. It clarified the cause of fetal OI in the subject family, provided guidance for any future pregnancy the couple might have and highlighted the value of WES in diagnosis of SD with unclear prenatal indications.

## Data Availability Statement

The datasets presented in this study can be found in online repositories. The names of the repository/repositories and accession number(s) can be found at: https://www.ncbi.nlm.nih.gov/, NG 011537.1; http://www.wwpdb.org/, NM_000935.

## Ethics Statement

The studies involving human participants were reviewed and approved by the Research Ethics Committee of the Shijiazhuang Obstetrics and Gynecology Hospital. The patients/participants provided their written informed consent to participate in this study.

## Author Contributions

QG designed the overall research strategy and supervised the whole process. JZ recruited the case, performed the prenatal counseling, sampling, and part of the genetic experiments. HH performed the other part of the genetic experiments and data analysis. WM and MY performed IHC. WC conducted protein modeling. DM performed prenatal ultrasonic exam. JZ and HH cooperated in the preparation of this manuscript. All authors contributed to the article and approved the submitted version.

## Conflict of Interest

The authors declare that the research was conducted in the absence of any commercial or financial relationships that could be construed as a potential conflict of interest.
