# Supplementary material for: Case Report: Exome Sequencing Identified a Novel Compound Heterozygous Variation in PLOD2 Causing Bruck Syndrome Type 2
Source: Front Genet. 2021 Feb 16;12:619948. doi: 10.3389/fgene.2021.619948 (PMC7921790; doi:10.3389/fgene.2021.619948)
Supplement: Supplementary file 1 [file Table_1.DOC]

**Table 1. *PLOD2* variants associating with BRKS2-like phenotype reported in literature**

| No. | GENE | EXON | cDNA | PROTEIN | DIAGNOSIS | PMID |
| --- | --- | --- | --- | --- | --- | --- |
| 1 | PLOD2 | 4 | c.497C>G | p.S166* | Kyphomelic dysplasia | 29178448 |
| 2 | PLOD2 | IVS4 | c.503-2A>G |  | Osteogenesis imperfecta IV | 28725987 |
| 3 | PLOD2 | 5 | c.517G>C | p.A173P | Bruck syndrome | 31001443 |
| 4 | PLOD2 | 8 | c.797G>T | p.G266V | BRKS2 | 15523624 |
| 5 | PLOD2 | 11 | c.1138C>T | p.R380C | Bruck syndrome | 29177700 |
| 6 | PLOD2 | 11 | c.1153T>C | p.C385R | Bruck syndrome | 29177700 |
| 7 | PLOD2 | 12 | c.1280A>G | p.N427S | OI without contractures | 20354512 |
| 8 | PLOD2 | IVS12 | c.1358+5G>A |  | Bruck syndrome | 22689593 |
| 9 | PLOD2 | 13 | c.1406G>A | p.G469E | Osteogenesis imperfecta III | 27509835 |
| 10 | PLOD2 | 15 | c.1559dupC | p.V523Cfs*7 | Bruck syndrome | 22689593 |
| 11 | PLOD2 | 15 | c.1624delT | p.Y542Tfs*25 | Bruck syndrome | 25238597 |
| 12 | PLOD2 | 16 | c.1682G>A | p.W561* | Kyphomelic dysplasia | 29178448 |
| 13 | PLOD2 | 17 | c.1754A>T | p.D585V | Kyphomelic dysplasia | 29178448 |
| 14 | PLOD2 | 17 | c.1764G>T | p.W588C | Osteogenesis imperfecta | 29178448 |
| 15 | PLOD2 | 17 | c.1828T>C | p.W610R | Osteogenesis imperfecta | 28116328 |
| 16 | PLOD2 | 17 | c.1856G>A | p.R619H | Bruck syndrome | 12881513 |
| 17 | PLOD2 | 17 | c.1864G>T | p.G622C | Bruck syndrome | 22689593 |
| 18 | PLOD2 | 17 | c.1865G>T | p.G622V | Bruck syndrome | 15523624 |
| 19 | PLOD2 | 17 | c.1880T>C | p.V627A | Bruck syndrome | 25238597 |
| 20 | PLOD2 | 17 | c.1886C>T | p.T629I | Bruck syndrome | 12881513 |
| 21 | PLOD2 | 18 | c.1982G>A | p.G661D | Bruck syndrome | 29177700 |
| 22 | PLOD2 | 19 | c.2038C>T | p.R680* | Bruck syndrome | 29177700 |
| 23 | PLOD2 | 19 | c.2060A>G | p.H687R | Bruck syndrome | 29178448 |
| 24 | PLOD2 | IVS19 | c.2122-2A>G |  | Bruck syndrome | 22689593 |
| 25 | PLOD2 | 20 | c.2217T>A | p.H739Q | Osteogenesis imperfecta | 27509835 |
